# Visual and non-visual effects of the phosphor-free white LED lamps rich in the 535–589-nm yellow-green light

**DOI:** 10.1038/s41377-025-01896-w

**Published:** 2025-06-20

**Authors:** Shanshan Zeng, Ya Guo, Wentao Hao, Xin Luo, Xing Guo, Jianli Zhang, Jianqi Cai, Guangxu Wang

**Affiliations:** 1 Laboratory of Visual Health and Safety Protection, China National Institute of Standardization, Beijing, China; 2 National Institute of LED on Si Substrate, Nanchang University, Nanchang, China

**Keywords:** Inorganic LEDs, Biophotonics

## Abstract

Despite an increasing number of researches focused on the photo-biological effects of lighting on visual and non-visual health, insights into the phosphor-free white LED lamps with different spectral power distributions remain unclear. In this study, human factors experiments were conducted with 30 participants. Variations in ocular physiological parameters and melatonin circadian rhythms were assessed for participants in an ordinary lamp, a full-spectrum lamp, and four phosphor-free white LED lamps rich in the 535–589 nm yellow-green light. It was observed that ocular functions exhibited significantly higher improvements under the four phosphor-free LED lamps compared to the ordinary and full-spectrum lamps, indicating the advantageous effects of phosphor-free lamps rich in the 535–589 nm yellow-green light on both visual and non-visual health.

## Introduction

Indoor environments have been increasingly affecting human health due to lifestyle changes with extended indoor activity time. Among the various environmental conditions, indoor light is especially crucial in the development of green, healthy, and sustainable buildings^1–3^. Researchers have revealed light effects on visual perception, including visual acuity and spatial vision, and exerts non-visual effects on physiological circadian rhythms and psychological health^4–6^. Due to the adverse effects of poor lighting quality on visual comfort, psychological health, and physiological circadian rhythms, it is necessary to clarify the impact of lighting photometric parameters on health.

**Fig. 1:**
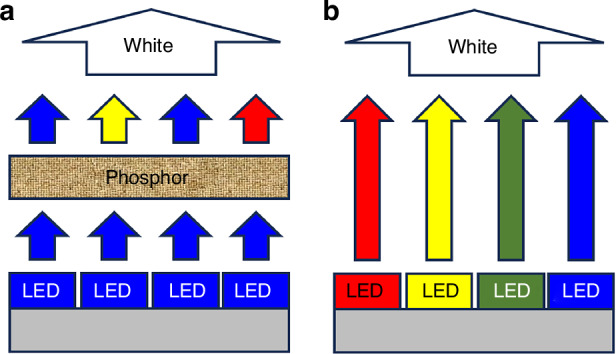
Two different white LED technical routes: **a** phosphor-converting LED and **b** phosphor-free LED

The photometric properties of indoor lighting, including illuminance^7,8^, uniformity^9^, luminance^10,11^, correlated color temperature (CCT)^12,13^, color rendering index (CRI)^12^, and glare^14^, are primarily determined by the light sources. Generally, there are two technical routes for white light LEDs used in indoor lighting: one is the conventional phosphor-converting (PC) technology, and the other is phosphor-free (PF) technology that is predicted to be the next generation of solid-state lighting technology. As shown in Fig. 1a, b, PC lamps contain blue LED chips and yttrium aluminum garnet (YAG) yellow phosphors, exhibiting the spectrum characterized by narrow blue crests and dispersive wavelength distributions in the green and red segments. As PC technology is based on electroluminescence and photoluminescence with down-conversion loss, phosphor quality degradation caused by heat accumulation generally tends to induce high blue ratio^15^. Differently, PF lamps present separate narrow crests in blue, green, yellow and red segments, as this type of lamps are made of mixed monochromatic LEDs with different wavelengths. Free from photoluminescence conversion loss, PF lamps have better optical performances, such as adjustable spectrum, which can be applied in smart lighting and on-demand lighting^16^. According to a report of the U.S. Department of Energy (DOE BTO Solid-State Lighting Program, “2022 DOE SSL R&D Opportunities”), the ultimate luminous efficacy of PC and PF lamp is 250 lm/W and 336 lm/W. Therefore, PF lamps are superior to PC lamps in green and healthy lighting.

In the visible wavelength segment, human eye is especially sensitive to yellow-green light, which contributes to the luminous flux and plays a crucial role in PF lighting technology. Nevertheless, the optical performance of PF lamp is limited by the low optical power of the yellow-emitting quantum wells (QWs) due to the poor quality of In-rich InGaN layer. Recently, researchers have made a significant achievement by improving the light efficiency of yellow LED, promoting the extensive application of PF lamps^17,18^. However, the superiority of PF to PC lamps in health-oriented lighting is unclear, as few researches have explored the effects of PF lamps on visual and non-visual health.

PF lamps differ from PC lamps in spectral power distribution (SPD), especially in the ratios of green, yellow, and red segments. Among these segments, red light has been discovered to be significant to human health due to its thermal effect and penetration feature^19^. Recently, researchers discovered that the 605–635-nm red light could promote melatonin secretion^19^. Compared to the red light, the green and yellow light has not been researched sufficiently. In this study, we compared the effects of PC and PF lamps on ocular functions and melatonin secretion, and discussed the potential applications of PF lamp in the field of health-oriented lighting and displays.

## Results

### Optical performances of experimental lamps

**Fig. 2:**
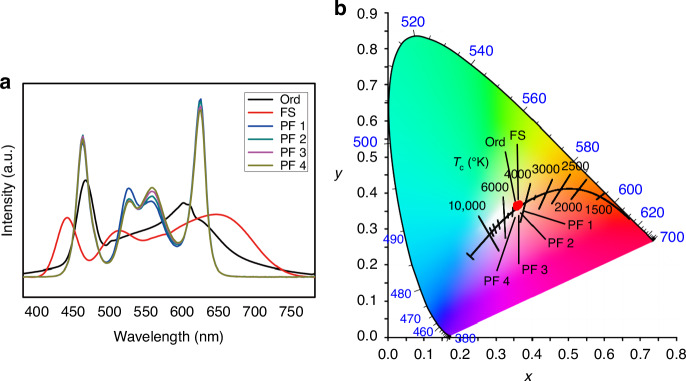
**a** The SPDs and **b** the chromaticity coordinates of the six experimental lamps including Ord, FS, PF 1, PF 2, PF 3, and PF 4

In the current study, experiments were performed using two PC lamps (including an ordinary lamp and a full-spectrum lamp, denoted as Ord and FS respectively) and four PF lamps (denoted as PF 1–PF 4). All the six lamps were side-glowing classroom lamp, with similar CCTs (approximately 4500 ± 250 K). During experiments, each lamp was mounted in the ceiling board. Adhering to the ISO 8995 (GB 50034-2013) standards and our prior research, the desk illuminance and uniformity were set approximately 500 lux and above 0.8 respectively. SPDs and chromaticity coordinates of the six experimental lamps (Ord, FS, PF 1, PF 2, PF 3, and PF 4) are shown in Fig. 2a, b. As shown in Fig. 2a, the four PF lamps are characterized by high ratios in the 535–589 nm yellow-green light.

### Variations of ocular functional parameters

During the human factor experiments, variations of ocular functional parameters were used to reflect the visual effects of the six experimental lamps. The ocular functional parameters contained ciliary accommodation (ACC), modulation transfer function (MTF), and the 12th-term high order aberrations (HOA12). ACC describes the refractive difference between the maximum and the minimum accommodative status of ciliary fibers, and is generally applied in the assessment of visual fatigue and myopia risk. MTF reflects the contrast ratio of outputs to inputs, while HOA12 indicates the dispersive spot caused by different refractive powers between central and peripheral lens. MTF and HOA12 are generally applied in the evaluation of vision quality.

Apart from the variations of ocular functional parameters (denoted as ∆ACC, ∆MTF, and ∆HOA12), we calculated the visual comfort (VICO) index according to the GB/T 44441 standard. Although the value of VICO reflects the level of visual fatigue, with smaller value corresponding to lower fatigue level, it is derived from objective physiological parameters, which avoids subjective biases and offers advantages over single-parameter assessments. Among the three variations of ocular functional parameters, ∆ACC describes the fatigue level of ciliary power, while ∆MTF and ∆HOA12 describe the reduction of vision quality. Larger variations of ocular functional parameters indicate higher levels of ocular fatigue and more effects of the lamps.

**Fig. 3:**
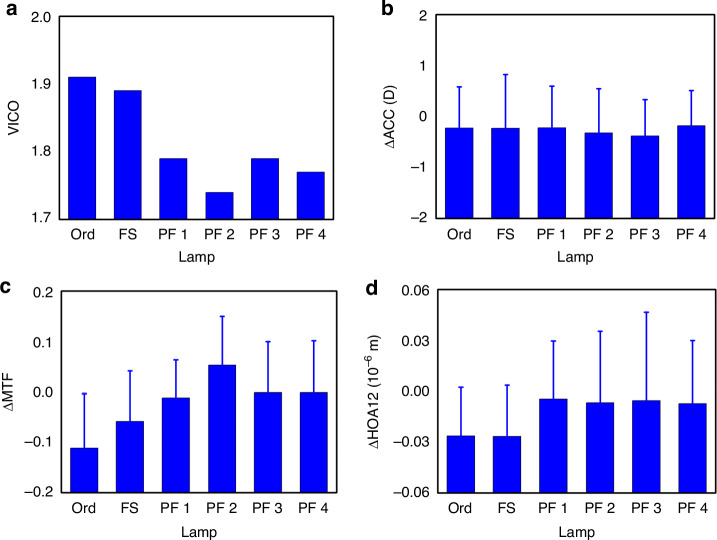
Values of **a** VICO, **b** ∆ACC, **c** ∆MTF, and **d** ∆HOA12 in different lamps (data are shown as mean ± SEM; *N* = 30)

The VICO indexes of the six experimental lamps are shown in Fig. 3a. The four PF LED lamps presented lower VICO values than the Ord and the FS lamps, implying that the four PF LED lamps caused less visual fatigue and better visual comfort compared to the Ord and the FS lamps. As shown in Fig. 3a, the VICO values of the four PF LED lamps were below 1.8, while those of the Ord and the FS lamps were close to 1.9. Among the four PF LED lamps, PF 2 stands out with the lowest VICO value, suggesting its superiority to other lamps in minimizing ocular fatigue.

∆ACC, ∆MTF, and ∆HOA12 were calculated by subtracting the 45^th^-min values with the 0^th^-min values. As shown in Fig. 3b–d and Supplementary Table S1, ANOVA results indicate that the ∆ACC values of the six experimental lamps were similar (*p* > 0 between any two lamps), while significant distinctions were observed in ∆MTF (*p* = 0.001 between Ord and the four PF lamps; *p* = 0.031 between FS and PF 1; *p* = 0.001 between FS and PF 2; *p* = 0.024 between FS and PF 3; *p* = 0.023 between FS and PF 4) and ∆HOA12 (*p* = 0.028 between Ord and PF 1; *p* = 0.048 between Ord and PF 2; *p* = 0.036 between Ord and PF 3; *p* = 0.054 between Ord and PF 4; *p* = 0.026 between FS and PF 1; *p* = 0.045 between FS and PF 2; *p* = 0.034 between FS and PF 3; *p* = 0.050 between FS and PF 4) between the PC and PF lamps. ACC describes the refractive power of crystalline lens which is connected to ciliary zonules, and ∆ACC is sensitive to photon amount. As the six experimental lamps had similar illuminances, the corresponding ∆ACC values for the six lamps presented no significant differences. MTF and HOA12 reflects vision quality, which is sensitive to photon wavelength characterization. Although the six experimental lamps had similar CCT and CRI, their SPDs are distinct.

∆MTF was positive for PF 2 yet negative for the other lamps, indicating that MTF functions were increased in PF 2 yet decreased in other lamps during the 45-min visual tasks. ∆HOA12 values were negative for all the lamps, suggesting that HOA12 functions were reduced in all the lamps during the 45-min visual tasks. According to the results of ∆MTF and ∆HOA12, the PF LED lamps rich in the 535–589-nm yellow-green light are likely to effectively alleviate functional reductions in vision quality compared to PC lamps. These improvements indicated the potential applications of PF lamps in the field of health-oriented lighting.

As HbO2 cortical blood-oxygen signals describe cortical activations, the correlations of the signals in different cortical areas indicate their functional connectivity levels. In this study, we selected three target areas: Point O locates in the primary visual cortex, close to the occipital bone; Points A and B locate in the dorsal and ventral pathways respectively. Therefore, O-A connectivity indicates visual signal transmission in the dorsal pathway, while O-B infers ventral pathway activities^20^. During the 45-min visual tasks, the variations of O-A and O-B connectivity were denoted as ∆O-A and ∆O-B respectively, describing the effects of the six experimental lamps on the activations of the dorsal and ventral pathways.

**Fig. 4:**
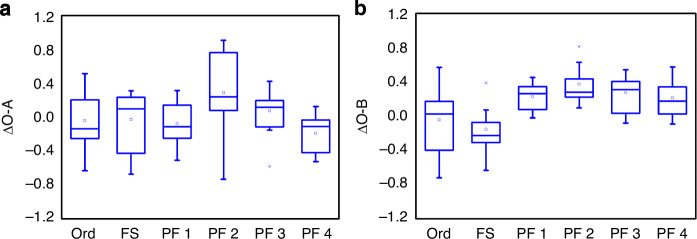
**a** ∆O-A and **b** ∆O-B correlations of participants in different lamps

As shown in Fig. 4a, b and Supplementary Table S2, ANOVA results indicate ∆O-A values were similar among the six experimental lamps. Overall, there were almost no differences between PC and PF LED lamps in ∆O-A. Differently, ∆O-B values were significantly higher in the four PF LED lamps than in the two PC lamps (*p* = 0.001 between Ord and PF 1; *p* = 0.001 between Ord and PF 2; *p* = 0.003 between Ord and PF 3; *p* = 0.002 between Ord and PF 4; *p* = 0.010 between FS and PF 1; *p* = 0.024 between FS and PF 2; *p* = 0.073 between FS and PF 3; *p* = 0.068 between FS and PF 4). The results implied that PF LED lamps rich in the 535–589-nm yellow-green light benefit the activation of the ventral pathway, which influence the visual perception regarding shape and color. Given that ∆MTF, ∆HOA12, and ∆O-B were all enhanced by the PF LED lamps, there might be certain relationship among these three parameters.

### Melatonin secretion

**Fig. 5:**
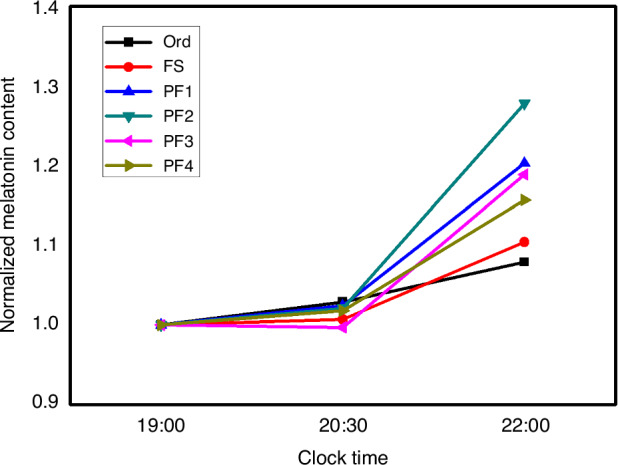
Normalized melatonin contents at 19:00, 20:30, and 22:00 in Ord, FS, PF 1, PF 2, PF 3, and PF 4 respectively (*N* = 30)

As shown in Fig. 5 and Supplementary Table S3, results of paired samples t-test indicate the melatonin contents in the six experimental lamps were similar from 19:00 to 20:30, yet different from 20:30 to 22:00, as the four PF LED lamps presented higher melatonin contents (*p* = 0.001 for PF 1; *p* < 0.001 for PF 2 and PF 3; *p* = 0.006 for PF 4). As normal circadian rhythm is characterized by low melatonin content in the daytime while high content at night, higher melatonin content at 22:00 than at 19:00 infers better circadian rhythm. Our results implied that the four PF LED lamps rich in the 535–589-nm yellow-green light were better for circadian rhythms compared to the two PC lamps.

## Discussion

The four PF LED lamps differed from the two PC LED lamps in SPD, as they presented significantly higher ratios in the segments of the 535–589-nm green and yellow light and the 605–635-nm red light.

Our previous work implied that the 605–635-nm red light had compensation effects on melatonin secretion^19^. Therefore, the beneficial effects of the four PF LED lamps on melatonin secretion seemed to result from their high ratios in the 605–635-nm red light. Nevertheless, excessively high ratios in the 605–635-nm red light are likely to enhance the melatonin contents in the daytime and induce drowsiness, disrupting normal circadian rhythm. According to our findings in this study, increases of melatonin contents from 20:30 to 22:00 were more significant in PF LED lamps (*p* = 0.001 for PF 1; *p* < 0.001 for PF 2 and PF 3; *p* = 0.006 for PF 4) than in PC LED lamps (*p* = 0.269 for Ord; *p* = 0.045 for FS), while from 19:00 to 20:30 there were almost no differences between the PF and PC LED lamps (*p* = 0.518 for Ord; *p* = 0.888 for FS; *p* = 0.622 for PF 1; *p* = 0.678 for PF 2; *p* = 0.946 for PF 3; *p* = 0.703 for PF 4), implying that the superiority of PF LED lamps to PC LED lamps resulted from not only the 605-635-nm red light but also the 535-589-nm green and yellow light. It seems that the combination of the 535–589-nm and the 605–635-nm light could make melatonin secretion normal, rather than simply improving melatonin contents.

For the S-, M-, and L- photoreceptor cone cells in retina, the photo sensitivity peaks are 420 nm, 535 nm, and 565 nm respectively. Compared to the two PC LED lamps, the four PF LED lamps could stimulate the photoreceptor cells more evenly due to the high ratios in the 535–589-nm green and yellow light.

## Materials and methods

### Light sources

In our study, the Ord and FS lamps were constructed with the ordinary and full-spectrum LED beads bought in the market, while the side-emitting classroom PF lamps (PF1-PF4, with different yellow-green-light ratios yet identical CCTs) were made of 3535 RGBY phosphor-free ceramic packaged LEDs, which were developed by the National Institute of LED on Silicon Substrate at Nanchang University through process of die attach, wire bond, and moulding. The SPDs and various modes of the 3535 RGBY phosphor-free ceramic packaged LEDs are shown in Supplementary Figs. S1 and S2 respectively. The epitaxial material of the yellow LED was fabricated using a Metal-Organic Chemical Vapor Deposition (MOCVD) instrument, growing high-quality InGaN quantum wells (QWs), enhancing carrier injection and radiative recombination efficiency^21–23^. The wall plug efficiency and luminous efficiency of the yellow LED are 32.8% and 200 lm/W, respectively, at the current density of 20 A/cm^2^ with a dominant wavelength of 566 nm and a half-peak width of 38 nm, while at the current density of 1 A/cm^2^, the wall plug efficiency and luminous efficiency are 51.6% and 318 lm/W, respectively, with a dominant wavelength of 564 nm and a half-peak width of 36 nm.

Adhering to ISO 8995 (GB/T 50034-2024) standards and our prior research, we set the lighting illuminance and Correlated Color Temperature (CCT) to be 500 lux and 4500 ± 250 K, respectively. The light environment is shown in detailed in Supplementary Table S4. Illuminance levels were measured using a luxmeter, and the measurement was conducted according to the standards JJG 245-2005 and GB/T 5700-2023. To ensure the amount of light entering eyes of each participant, the sitting eye height was controlled to the same level (1.2 m) by adjusting the chair height, all measurements adhering to the standard GB/T 5703-2023. As participants’ sights fluctuated within the area of the A4 paper at the desk center, the difference of eye level illuminances among participants was deemed to be low enough and negligible.

### Human factor experiments

In this study, human factor experiments were performed on 30 participants using the six experimental lamps. None of them had oculopathies or habits of consuming alcohol, sleeping pills, caffeine, or hormone-related drugs. Their detailed information is provided in Supplementary Table S5.

The experiment for each lamp lasted for a week, therefore the entire experimental duration was six weeks. Participants worked and lived in the light environment of the corresponding lamp from Monday to Wednesday without being told which lamp they were using each time, and restored in their daily-used lamps from Thursday to Sunday. The lamps used from Thursday to Sunday were dorm lamps in the same model. Each participant was required to finish supper before 18:00 and go to bed at 22:30.

Visual effects were investigated on Wednesday by arranging participants to perform visual tasks, which comprised a 20-min Landolt-rings counting and a 25-min color recognition segments, which covered primary visual tasks in daily life. Ocular physiological parameters, including ACC, MTF, and HOA12 (the 12th order aberration), were recorded before and after these tasks. Cortical functional measurements were conducted on a subset of participants: 12 participants whose cortical NIR signals were strong enough for cortical functional analysis were selected from the 30 participants. During the 45-min visual task, participants wore electrode cap for the collection of near-infrared (NIR) blood-oxygen signal.

**Fig. 6:**
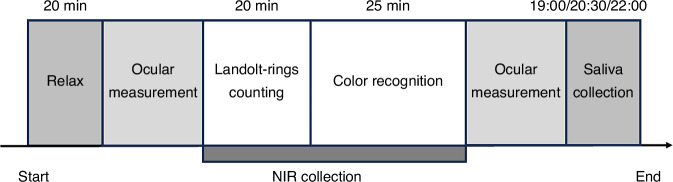
The entire process of the human factor experiment

Non-visual effects were explored by collecting saliva at 19:00, 20:30, and 22:00 on Wednesday for each participant, and saliva melatonin contents were then measured. Participants had supper before 18:00, and then gargled with pure water. Moreover, participants were not allowed to eat within 30 min before saliva collection, and forbidden to drink water within 10 min before saliva collection. The entire process was shown in Fig. 6.

### Measurement

ACC was collected by the NIDEK AR-1S Auto Refractometer; MTF and HOA12 were analyzed through the NIDEK OPD-Scan III Refractive Power & Corneal Analyzer. During the measurement, participants were seated in front of the instrument, staring at the specified area of the instrument. Visual functional data were collected by the instrument and transported to the computer. For ACC, MTF, and HOA12, we subtracted the 45th-min values with the initial values, and obtained the variations of these three parameters (denoted as ∆ACC, ∆MTF, and ∆HOA12). According to the standard GB/T 44441, we calculated the VICO index for each lamp.

Visual cortical blood-oxygen signals were analyzed by measuring the intensities of 690-nm and 830-nm wavelength light and performing hemodynamics calculation. The instrument was the Artinis NIRS system equipped with 20 detectors. Electrodes distribution was shown in Supplementary Fig. S3. Visual stimulation and signal collection were executed synchronously. To reduce possible disturbs, we chose the data from the 5th min to the 5.5th min (30-s data), and the data from the 40th min to the 40.5th min (another 30-s data) for analysis.

We used the nirsLAB software (NIRX Co. Ltd.) to analyze fNIR data. We excluded the channels with the enhancement factor above 0.8 and the variation coefficient above 15%. Motion artifact correction was performed using functions “Remove Discontinuities” and “Remove Spike artifacts”. We also performed 0.01–0.2 Hz bandpass filtering.

For saliva collection, each participant kept a piece of cotton under the tongue, and after 1 min the cotton was extracted from the mouth with tweezers and stored at −20 °C with the SARSTEDT Salivette tube (Germany). Saliva collections were performed four times with the interval more than 30 min. Melatonin contents were measured using the Enzyme-Linked ImmunoSorbent Assay (ELISA) analysis.

## Supplementary information

Supplementary Information

## Data Availability

The data in this study are available on request from the corresponding author upon reasonable request.
