# Supplementary material for: Visual and non-visual effects of the phosphor-free white LED lamps rich in the 535–589-nm yellow-green light
Source: Light Sci Appl. 2025 Jun 20;14:219. doi: 10.1038/s41377-025-01896-w (PMC12181328; doi:10.1038/s41377-025-01896-w)
Supplement: Supplementary file 1 — Supplementary Information [file 41377_2025_1896_MOESM1_ESM.docx]

**Supplementary Information for**

**Visual and Non-visual Effects of the Phosphor-Free White LED Lamps Rich in the 535-589-nm Yellow-Green Light**

Shanshan Zeng,^1^ Ya Guo,^1^ Wentao Hao,^1^ Xin Luo,^2^ Xing Guo,^2^ Jianli Zhang,^2^ Jianqi Cai,^1^* Guangxu Wang^2^*

Affiliations

^1^ Laboratory of Visual Health and Safety Protection, China National Institute of Standardization, Beijing, 100191, China.

^2^ National Institute of LED on Si Substrate, Nanchang University, Nanchang 330096, China.

* Corresponding author Email: [caijq@cnis.ac.cn](mailto:caijq@cnis.ac.cn); guangxuwang@ncu.edu.cn

Table S1 ANOVA p values of ∆ACC, ∆MTF, and ∆HOA12 in different lamps (N=30); the Dunnett method was used in ANOVA

| Comparison | | ∆ACC | ∆MTF | ∆HOA12 |
| --- | --- | --- | --- | --- |
| Ord | FS | 0.986 | 0.034 | 0.978 |
|  | PF 1 | 0.981 | 0.001 | 0.028 |
|  | PF 2 | 0.657 | 0.001 | 0.048 |
|  | PF 3 | 0.475 | 0.001 | 0.036 |
|  | PF 4 | 0.826 | 0.001 | 0.054 |
| FS | Ord | 0.986 | 0.034 | 0.978 |
|  | PF 1 | 0.968 | 0.031 | 0.026 |
|  | PF 2 | 0.670 | 0.001 | 0.045 |
|  | PF 3 | 0.486 | 0.024 | 0.034 |
|  | PF 4 | 0.812 | 0.023 | 0.050 |
| PF 1 | Ord | 0.981 | 0.001 | 0.028 |
|  | FS | 0.968 | 0.031 | 0.026 |
|  | PF 2 | 0.640 | 0.023 | 0.822 |
|  | PF 3 | 0.461 | 0.917 | 0.916 |
|  | PF 4 | 0.844 | 0.912 | 0.781 |
| PF 2 | Ord | 0.657 | 0.001 | 0.048 |
|  | FS | 0.670 | 0.001 | 0.045 |
|  | PF 1 | 0.640 | 0.023 | 0.822 |
|  | PF 3 | 0.787 | 0.030 | 0.905 |
|  | PF 4 | 0.507 | 0.030 | 0.958 |
| PF 3 | Ord | 0.475 | 0.001 | 0.036 |
|  | FS | 0.486 | 0.024 | 0.034 |
|  | PF 1 | 0.461 | 0.917 | 0.916 |
|  | PF 2 | 0.787 | 0.030 | 0.905 |
|  | PF 4 | 0.351 | 0.995 | 0.863 |
| PF 4 | Ord | 0.826 | 0.001 | 0.054 |
|  | FS | 0.812 | 0.023 | 0.050 |
|  | PF 1 | 0.844 | 0.912 | 0.781 |
|  | PF 2 | 0.507 | 0.030 | 0.958 |
|  | PF 3 | 0.351 | 0.995 | 0.863 |

Table S2 ANOVA p values of ∆O-A and ∆O-B in different lamps; the Dunnett method was used in ANOVA

| Comparison | | O-A | O-B |
| --- | --- | --- | --- |
| Ord | FS | 0.978 | 0.038 |
|  | PF 1 | 0.792 | 0.001 |
|  | PF 2 | 0.552 | 0.001 |
|  | PF 3 | 0.395 | 0.003 |
|  | PF 4 | 0.467 | 0.002 |
| FS | Ord | 0.978 | 0.038 |
|  | PF 1 | 0.982 | 0.010 |
|  | PF 2 | 0.836 | 0.024 |
|  | PF 3 | 0.608 | 0.073 |
|  | PF 4 | 0.721 | 0.068 |
| PF 1 | Ord | 0.792 | 0.001 |
|  | FS | 0.982 | 0.010 |
|  | PF 2 | 0.997 | 0.995 |
|  | PF 3 | 0.965 | 0.350 |
|  | PF 4 | 0.991 | 0.459 |
| PF 2 | Ord | 0.552 | 0.001 |
|  | FS | 0.836 | 0.024 |
|  | PF 1 | 0.997 | 0.995 |
|  | PF 3 | 0.999 | 0.667 |
|  | PF 4 | 0.999 | 0.785 |
| PF 3 | Ord | 0.395 | 0.003 |
|  | FS | 0.608 | 0.073 |
|  | PF 1 | 0.965 | 0.350 |
|  | PF 2 | 0.999 | 0.667 |
|  | PF 4 | 0.994 | 0.999 |
| PF 4 | Ord | 0.467 | 0.002 |
|  | FS | 0.721 | 0.068 |
|  | PF 1 | 0.991 | 0.459 |
|  | PF 2 | 0.999 | 0.785 |
|  | PF 3 | 0.994 | 0.999 |

Table S3 Melatonin content differences at 19:00, 20:30, and 22:00 in various lamps (N=30), analyzed using paired samples t-test

| Melatonin Content | 19:00 and 20:30 | 20:30 and 22:00 |
| --- | --- | --- |
| Ord | t=-0.654, p=0.518 | t=-1.128, p=0.269 |
| FS | t=-0.142, p=0.888 | t=-2.094, p=0.045 |
| PF 1 | t=-0.498, p=0.622 | t=-3.750, p=0.001 |
| PF 2 | t=-0.420, p=0.678 | t=-5.192, p<0.001 |
| PF 3 | t=0.069, p=0.946 | t=-4.234, p<0.001 |
| PF 4 | t=-0.385, p=0.703 | t=-2.945, p=0.006 |

Table S4 Photometric parameters of the Ord, FS, PF 1, PF 2, PF 3, and PF 4

| Lamp | Illuminance (lux) | CCT (K) | CRI |
| --- | --- | --- | --- |
| Ord | 493.8 | 4500±250 | ≥ 90 |
| FS | 506.1 |  |  |
| PF 1 | 522.7 |  |  |
| PF 2 | 507.1 |  |  |
| PF 3 | 486.6 |  |  |
| PF 4 | 511.1 |  |  |

Table S5 Detailed information of participants

| Item | Information |
| --- | --- |
| Participant total number | - 30 |
| Age distribution | - 19~23 |
| Diopter distribution | - 40%: 0.50 D ~ -1.00 D - 30%: -1.00 D ~ -3.00 D - 30%: -3.00 D ~ -5.00 D |
| Visual acuity distribution | - 40%: 0.8 - 60%: 1.0 |
| Anisometropia above 2.5 D | - None |
| Intraocular pressure range | - 14~20 |
| Sex | - 50%: Male - 50%: Female |


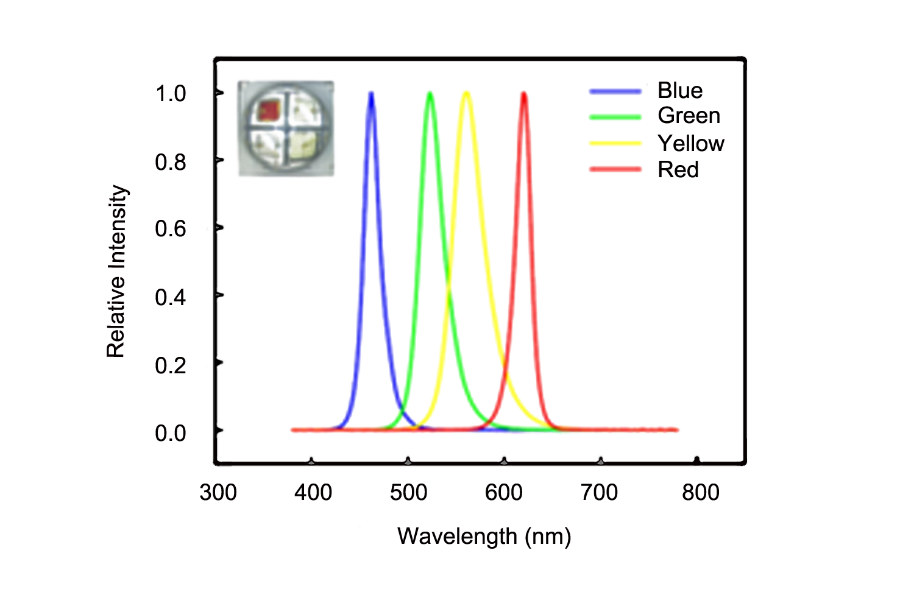


Fig. S1 Spectra of the 3535 RGBY, blue, green, yellow, and red LEDs


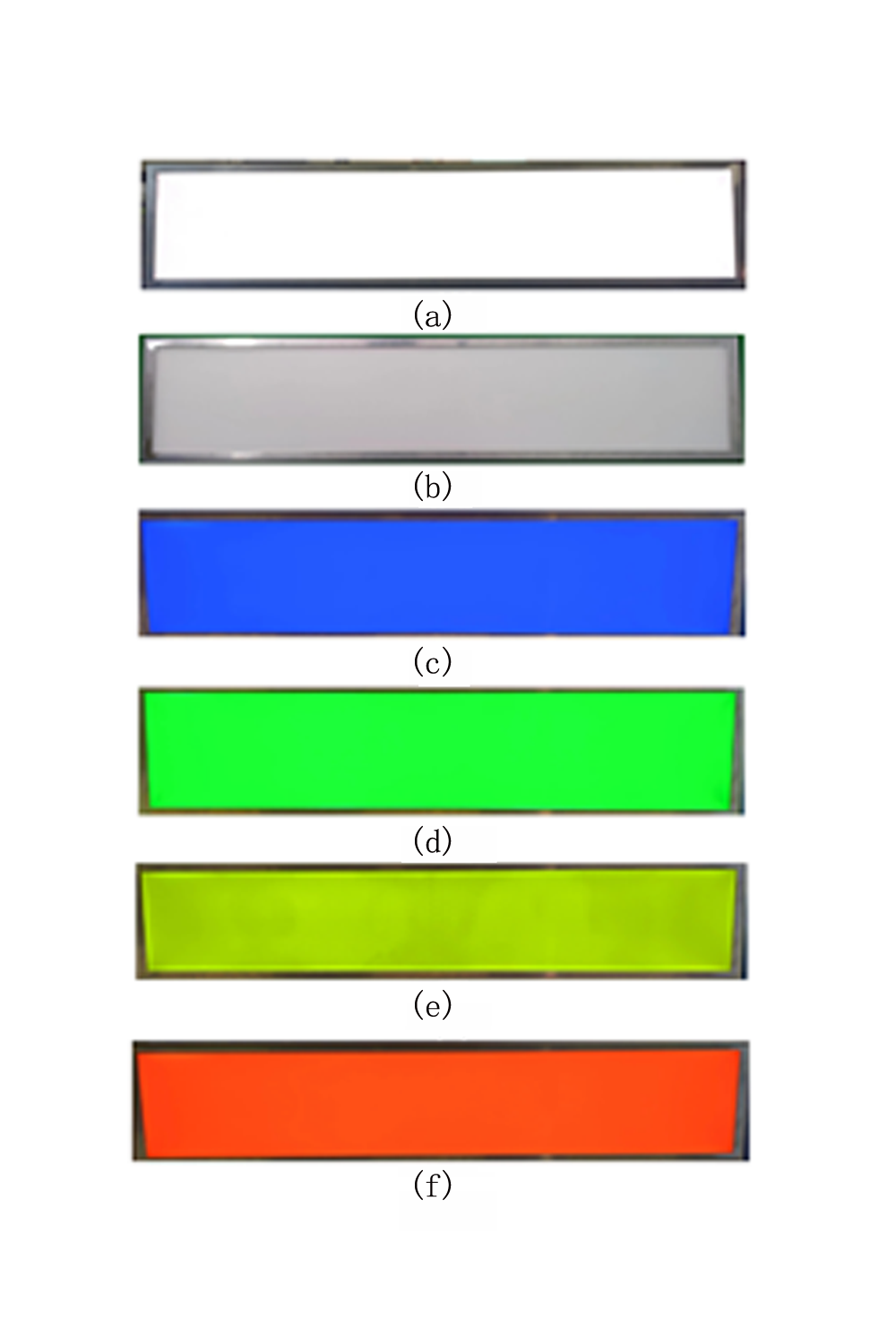


Fig. S2 RGBY-LED lamp in (a) darkness mode, (b) 4500±250 K mode, (c) blue monochromatic LED mode, (d) green monochromatic LED mode, (e) yellow monochromatic LED mode, (f) red monochromatic LED mode


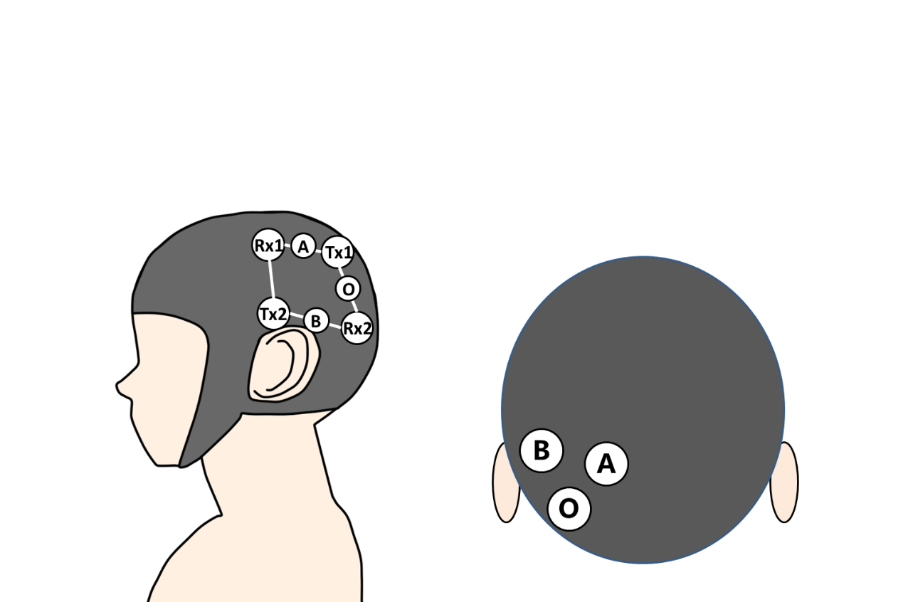


Fig. S3 Locations of O, A, and B for visual cortical blood-oxygen analysis
